# A Case of Chronic Eosinophilic Pneumonia Associated With the Use of Weight Loss Combination Medication Naltrexone-Bupropion

**DOI:** 10.7759/cureus.50621

**Published:** 2023-12-16

**Authors:** Anastasia Schuldt, Asif Najmuddin, Munish Sharma, Salim Surani

**Affiliations:** 1 Internal Medicine, University of Kentucky College of Medicine, Lexington, USA; 2 Internal Medicine, The Medical Center at Bowling Green, Bowling Green, USA; 3 Pulmonary and Critical Care, Baylor Scott & White Medical Center - Temple, Temple, USA; 4 Medicine, Texas A&M University, College Station, USA

**Keywords:** pneumonia, eosinophil, bupropion, naltrexone, eosinophilic pneumonia

## Abstract

Chronic eosinophilic pneumonia (CEP) is not a commonly encountered pulmonary disease that presents with bilateral pulmonary infiltrates accompanied by peripheral and bronchoalveolar lavage (BAL) eosinophilia. Recovery is rapid with systemic steroids but has frequent recurrences. We present a case with the classic presentation of CEP that appears to be related to a weight loss medication called naltrexone-bupropion. This case is unique in that this drug combination does not appear to have an established link to CEP, though literature reveals possible association with its individual components. Understanding the mechanism underlying this link may help to better understand CEP as a disease process.

## Introduction

Chronic eosinophilic pneumonia (CEP) is not a very commonly encountered pulmonary disease that is often characterized by dyspnea and cough accompanied by bilateral pulmonary infiltrates and peripheral eosinophilia. This disease often presents in middle-aged women, often nonsmokers, with a history of atopic diseases such as asthma or eczema. It is estimated to account for up to 2.5% of cases of all interstitial lung diseases [1]. Although sputum analysis is often unrevealing, bronchoalveolar lavage (BAL) with eosinophilia of >25% is diagnostic. Lung biopsy is usually unnecessary and reserved for cases with a questionable diagnosis. Often initially mistaken for pneumonia, CEP rapidly remits following systemic steroid treatment but has a high rate of recurrence [2].

We present a patient with classic CEP in terms of epidemiology and clinical presentation. However, the onset of CEP seems to correlate with the initiation of naltrexone-bupropion combination medication, used by the patient to assist with weight loss. The literature review suggests prior rare case reports of CEP associated with intramuscular naltrexone [3,4] and eosinophilia associated with bupropion [5].

This case is notable for two important reasons. The first is to establish a possible correlation between the use of naltrexone-bupropion and the development of CEP, as it would be interesting to uncover other cases in the future. The second is to emphasize the importance of risk analysis, as diagnosis could be established with BAL alone, while our patient underwent concurrent lung biopsy and suffered significant hemoptysis leading to intubation with ICU transfer and prolongation of hospital stay.

## Case presentation

A 42-year-old African American woman presented to the emergency department for a week with exertional dyspnea and a non-productive cough. Over the last six months, she had been diagnosed with recurrent episodes of pneumonia, which was treated on an outpatient basis with antibiotics, steroids, and inhaled bronchodilators. Her symptoms would temporarily improve and then recur. Five days before the presentation, she was diagnosed again with pneumonia in an urgent care clinic and started on cefdinir. She had been otherwise healthy and had no comorbidities except a history of iron deficiency anaemia. Further questioning revealed that she had started taking an appetite suppressant, naltrexone/bupropion, shortly before the repeated episodes of pneumonia began. She denied any recent lifestyle changes, did not smoke, and worked as an occupational therapist.

Vital signs were notable for mild tachycardia, tachypnea, and hypoxemia, which improved on supplemental oxygen. A physical exam revealed right-sided crackles and rhonchi but was otherwise unremarkable. A complete blood count (CBC) revealed leukocytosis with eosinophilia (Table 1).

**Table 1:** Complete blood count results showing leukocytosis and eosinophilia.

	Complete blood count results	Reference range
Total white blood cell counts	19.6	4.8-10.8 10*9 /L
Neutrophils	35%	40-60%
Lymphocytes	4%	20-40%
Eosinophils	58%	1-4%

**Figure 1:**
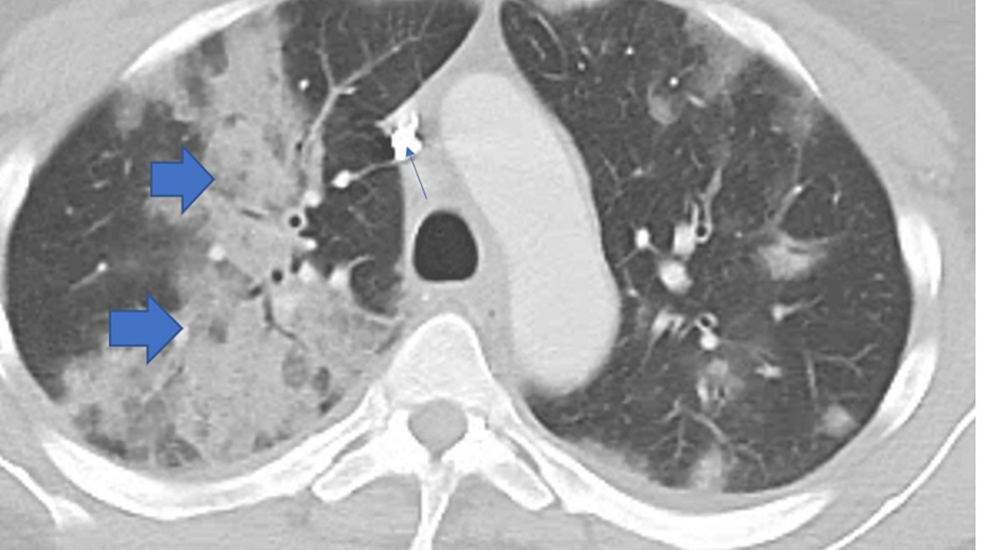
Computed tomography of the chest showing bilateral airspace disease (bold blue arrows on right-sided findings).

**Figure 2:**
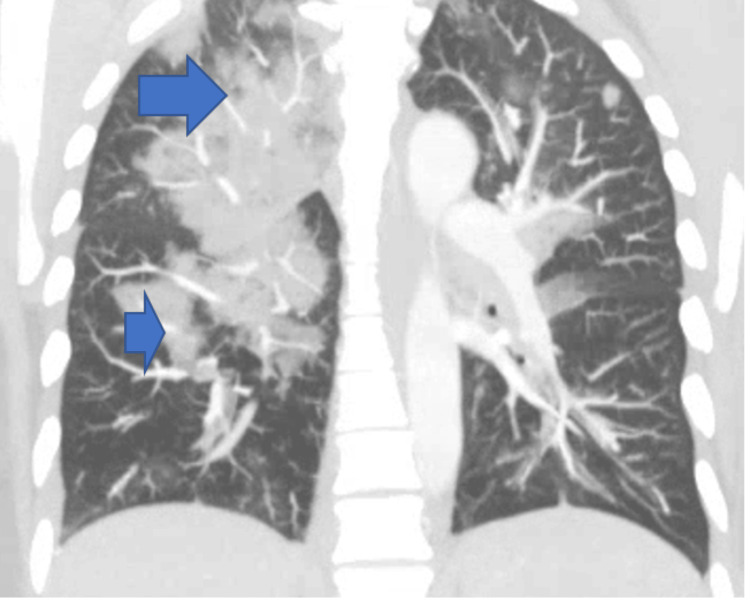
Computed tomography of the chest (sagittal section) showing bilateral airspace disease (bold blue arrows on right-sided findings).

Despite a history of iron deficiency anemia, she was not anemic on presentation. She remarked that her hematologist had noted elevated eosinophils on her most recent CBC, but no cause was identified at that time. Computed tomography (CT) angiogram of the chest was negative for pulmonary embolism but elucidated bilateral airspace disease with mediastinal and hilar lymphadenopathy. Consolidative changes are shown by bold blue arrows in the CT chest imaging (Figures 1-2).

She was started on empiric antibiotics for presumed community-acquired pneumonia. She underwent bronchoscopy with BAL and transbronchial biopsy of the right upper lobe but experienced significant hypoxemia because of bleeding during the biopsy and required intubation. Following the biopsy, she received systemic steroid therapy. BAL revealed eosinophilic predominance (75% of nucleated cells) with culture-negative bacteria, acid-fast bacilli, and fungi. Lung biopsy revealed eosinophilic pneumonia with interstitial eosinophils and chronic inflammatory cells, negative for bacteria on culture. Pathology was negative for parasites and did not indicate vasculitis. A set of blood cultures were negative.

The morning following the biopsy, the patient was extubated and did not experience hemoptysis or further hypoxemia. She continued to improve clinically with resolving consolidation on the chest X-ray. She was discharged on hospital day four on oral steroid therapy prednisone that was initially started at 0.5 mg/kg/day. The initial dose of prednisone was continued for two weeks after the complete resolution of symptoms and chest radiography abnormality. Prednisone was gradually tapered off in the next four weeks thereafter. She was advised to discontinue the use of naltrexone/bupropion, which was the suspected trigger of her eosinophilic pneumonia. Subsequent follow-up revealed sustained resolution of symptoms and peripheral eosinophilia even when she was taken off systemic steroids.

## Discussion

The case described above is the classic presentation of CEP. Though a relatively rare condition, CEP typically presents with persistent dyspnea, dry or productive cough, fever, and night sweats. Due to this disease's prolonged nature and rarity, CEP often takes months of symptoms before a diagnosis is made. The physical exam is variable, with one-third of patients demonstrating wheezing and another one-third having crackles. Chest radiograph demonstrates bilateral pulmonary opacities, often in the upper lobes and near the periphery, described as a “photographic negative of pulmonary edema” pattern. These opacities may even be migratory [1]. The first hint at CEP, in this case, came from peripheral eosinophilia on CBC. BAL is diagnostic, which almost exclusively reveals > 25% eosinophils. Sputum analysis is usually unhelpful and is often negative for eosinophils. Lung biopsy is usually unnecessary but can confirm the diagnosis of CEP in the rare instance of unrevealing BAL. Interstitial and alveolar eosinophils and histiocytes characterize lung biopsy [1]. These features are consistent with our case's BAL and lung biopsy findings. In our patient, a lung biopsy was performed concurrently with BAL, although the BAL findings ultimately were sufficient to confirm the diagnosis of CEP. In retrospect, lung biopsy, though also supportive of CEP, may not have been entirely necessary. This is a very important point to consider, as our patient suffered from post-biopsy bleeding and hypoxemia requiring intubation.

Our case patient represents the typical demographics of CEP, with a female: male ratio of 2:1 and often presenting in the fourth or fifth decade of life. Patients who suffer from CEP are often nonsmokers but have atopic conditions such as asthma or eczema [1]. Differential diagnosis includes fungal infections (e.g., aspergillosis) and vasculitis (e.g., eosinophilic granulomatosis with polyangiitis, formerly Churg Strauss). Chest radiographs may appear like cryptogenic organizing pneumonia (COP), but COP does not have eosinophilia on BAL and has a slower recovery compared to CEP [1].

It is also important to note that CEP has some similarities to acute eosinophilic pneumonia (AEP), with some key differences in epidemiology and laboratory findings. Unlike CEP, AEP is characterized by a rapid symptom onset (< 1 month) and commonly occurs without peripheral eosinophilia [1]. AEP is similar to CEP in that patients are often atopic, chest X-ray shows bilateral infiltrates, BAL is diagnostic with >25% eosinophils, lung biopsy is not usually necessary, and the prognosis is excellent with rapid response to systemic steroid therapy [1,2]. AEP is frequently mistaken for pneumonia and often treated like acute respiratory distress syndrome (ARDS) until eosinophilia is revealed on BAL [2]. Whereas CEP often presents in a smoldering fashion in nonsmokers and often recurs with steroid discontinuation. AEP is strongly correlated to smoking or other inhalation exposures. Some evidence has suggested that AEP often resolves following smoking cessation or exposure removal - in some cases even without steroid treatment - but rapidly recurs upon re-exposure [2,6-7].

Given the correlation of symptoms in our patient to the start of naltrexone-bupropion treatment, this case is suspicious of a drug-induced etiology. The literature review is unrevealing for prior cases of eosinophilic pneumonia associated with a naltrexone-bupropion combination. Drugs that have an established connection to eosinophilic pneumonia include NSAIDs, methotrexate, cocaine, and antibiotics such as ampicillin and nitrofurantoin [1]. Cases of eosinophilic pneumonia have also been reported with some cephalosporins, including ceftaroline, cephalexin, and cephradine [8]. Given that our case patient was treated with antibiotics on several occasions for pneumonia prior to diagnosis, it is prudent to consider the possibility of a medication effect. However, it appears that her symptoms began after starting naltrexone-bupropion and prior to antibiotic treatment, suggesting that antibiotics were not the culprit. Furthermore, her symptoms would briefly resolve following antibiotics and steroids and worsen shortly thereafter, lending further suspicion to a non-antibiotic trigger.

A few cases of eosinophilic pneumonia have been reported with the use of long-acting intramuscular naltrexone. In one study, long-acting naltrexone (380 mg or 190 mg in microspheres) was used to treat alcohol dependence, given as an intramuscular injection every four weeks. Of 205 pts in the higher-dose group, there was one reported case of eosinophilic pneumonia and one case of interstitial pneumonia. This appears to be the first reported instance of these adverse effects in naltrexone microsphere use [3]. Another case describes eosinophilic pneumonia that developed in a 59-year-old man one month following the start of intramuscular naltrexone for alcohol dependence, with recovery following a change to oral naltrexone [4]. At least two other cases of intramuscular naltrexone-induced AEP have been described [9,10].

There have been a few cases of eosinophilia reported with bupropion treatment. In one example, a 48-year-old woman developed a dry cough and myalgias 19 days after starting bupropion (150 mg daily) for depression. CBC demonstrated eosinophilia with normal pre-treatment CBC. The authors cite at least two prior cases of eosinophilia associated with bupropion. Hematologic reactions to bupropion are relatively rare, the most common of which is leukopenia. In this case, the patient’s symptoms and eosinophilia resolved following discontinuation of bupropion [5]. In the setting of a dry cough, one may speculate whether there may have been an eosinophilic pneumonia component to this drug reaction, but this case does not mention any abnormal imaging studies or invasive testing.

Literature review at the time of this case report is unable to surface any prior link between combination naltrexone-bupropion and eosinophilic pneumonia; this association appears to be novel. However, given the prior rare instances of eosinophilic pneumonia in microsphere naltrexone and eosinophilia in bupropion use, it is possible that either of these medications, alone or in combination, may have contributed to the development of this disease in our patient. It would be interesting to observe whether future cases of CEP may be linked to this drug combination.

Our patient was managed with systemic steroids, inhaled steroids, and discontinuation of the suspected offending drug, naltrexone-bupropion. The high recurrence rate of CEP can lead to steroid dependence, provoking research into other possible treatment options. One case report outlines a woman who maintained remission on inhaled steroids following systemic steroid cessation [11], but further research is necessary to conclude whether inhaled steroids may contribute to remission maintenance. There are few case reports that have reported that the anti-immunoglobulin E (anti-IgE) antibody omalizumab was used to treat cases of steroid-dependent eosinophilic pneumonia [12,13]. In some cases, patients were able to maintain disease remission on inhaled corticosteroid/long-acting beta-agonist inhalers following omalizumab treatment [14]. These cases generally involved patients with atopic features such as asthma. However, research suggests that IgE does not directly lead to mast cell degranulation, the inflammatory signaling pathways enhanced by IgE lead to increased cytokine production and prolongation of mast cell life, which may contribute to increased systemic mast cell presence. This mechanism appears to be independent of allergen cross-linking [15].

## Conclusions

These cases suggest that there is still much to learn regarding the underlying pathophysiology of CEP, and management options will likely evolve as the scientific community learns more about this disease. The CEP needs to be considered when the clinical presentation is of CEP seen in the context of medication use.
